# An Interoperable Digital Twin with the IEEE 1451 Standards

**DOI:** 10.3390/s22197590

**Published:** 2022-10-07

**Authors:** Helbert da Rocha, João Pereira, Reza Abrishambaf, Antonio Espirito Santo

**Affiliations:** 1 Department of Electromechanical Engineering, University of Beira Interior, 6200-001 Covilhã, Portugal; 2 Instituto de Telecomunicações, Delegação da Covilhã, 1049-001 Lisboa, Portugal; 3 Department of Engineering Technology, Miami University, Hamilton, OH 45011, USA

**Keywords:** IEEE 1451 semantic interoperability, IEEE 1451 standards, semantic digital twin interoperability, industry 4.0

## Abstract

The shop floor or factory floor is the area inside a factory where manufacturing production is executed. The digitalisation of this area has been increasing in the last few years, introducing the Digital Twin (DT) and the Industry 4.0 concepts. A DT is the digital representation of a real object or an entire system. A DT includes a high diversity of components from different vendors that need to interact with each other efficiently. In most cases, the development of standards and protocols does not consider the need to operate with other standards and protocols, causing interoperability issues. Transducers (sensors and actuators) use the communication layer to exchange information with digital contra parts, and for this reason, the communication layer is one of the most relevant aspects of development. This paper covers DT development, going from the physical to the visualisation layer. The reference architecture models, standards, and protocols focus on interoperability to reach a syntactic level of communication between the IEEE 1451 and the IEC 61499 standards. A semantic communication layer connects transducer devices to the digital representation, achieving a semantic level of interoperability. This communication layer adds semantics to the communication process, allowing the development of an interoperable DT based on the IEEE 1451 standards. The DT presented reaches the syntactic and semantic levels of interoperability, allowing the monitoring and visualisation of a prototype system.

## 1. Introduction

Since the appearance of the Internet of Things (IoT) concept in the 2000s, the aim has been to establish communication between different devices from different vendors to contribute to achieving a goal [1]. With the Industrial IoT (IIoT) in North America and the Fourth Industrial Revolution (I4.0) in Germany, researchers are working to achieve the connectivity goal of smart devices connected through the internet to improve flexibility, quality, and productivity. IIoT allows the connection of all the elements inside a manufacturing process (e.g., devices, machines, and control systems) from the lower-level information systems to the business level. Collecting and analysing data will lead to an optimal industrial operation. In contrast, I4.0 addresses the production efficiency in smart factories by employing a Cyber-Physical System (CPS). The term CPS was first utilised in 2006, but the base concept of CPS has been developed since the 1970s [2,3]. A CPS requires that the physical asset have a digital representation, where models store the data, updating them in real-time, thereby turning them into a Digital Twin (DT) [4]. It can be combined with Artificial Intelligence (AI), Cloud Computing (CC), big data, edge computing, and wireless sensor networks for the digitalisation of different assets, systems, and processes inside an industry using data acquired from sensors [5].

The concept of the Digital Twin (DT) was employed by the National Aeronautics and Space Administration (NASA) in the 1960s as a “living model” on the Apollo spaceship program to mirror, simulate, and predict the condition between a space vehicle on earth and another one in the space [6]. However, the generic term “Digital Twin” was firstly introduced by Michael Grieves at the University of Michigan during a lecture in 2003 as a “digital equivalent to a physical product” [6,7,8]. In 2012, NASA defined a DT as follows: “A Digital Twin is an integrated multiphysics, multiscale, probabilistic simulation of an as-built vehicle or system that uses the best available physical models, sensor updates, fleet history, etc., to mirror the life of its corresponding flying twin” [9]. However, there are many other definitions for DTs [5,6,9,10,11].

DTs have been employed by industries and organisations in different scenarios, including health monitoring, agriculture, smart cities, smart grid, manufacturing, meteorology, education, and automotive [8]. In manufacturing, the DT supports the development of a production process, making it reliable, flexible, and predictable with real-time updates. At the same time and it is possible to visualise and monitor the processes [7]. Challenges addressed in developing a DT include, among others, security, real-time data delivery, sensor network, interoperability, etc. The development of reference architecture models standardised and focused on simplifying the CPS’s development, implementation, and interoperability solves most issues. The reference architecture models are the abstraction of the specified needs and technologies to develop a CPS, such as connectivity and communication, device management, data collection and analyses, scalability, and security, turning them into generic guidelines [12]. 

It is estimated that about 40% of the success of the IoT depends on addressing interoperability issues [13]. The unavailability of interoperability increases the cost and complexity of development and integration inside the IIoT [4]. Driving the interoperability is a key element for the IIoT and I4.0, and consequently for the DTs [13]. A DT must collect, aggregate, and exchange data and information between vendors, those different communications become an interoperability issue [11]. Different levels of interoperability are present inside the I4.0 communication layer. For a DT, the main requirement is to achieve the semantic levels by exchanging meaningful data ambiguously [14]. This level is obtained using a framework inside a reference architecture model, such as oneM2M, Open Platform Communications Unified Architecture (OPC UA), ontologies, and vocabularies [4,15].

The shop floor digitalisation is based on the data acquired from the sensors and actions sent to actuators, creating a digital representation of the elements placed on the factory floor. The IEEE 1451 family of standards was developed to manage transducers (sensors and actuators) in the physical layer. From the data acquisition to the exchange of data through the internet, the IEEE 1451 communication layer fulfils some of the requirements for the I4.0 [16]. Combined with the IEC 61499 standard for control and data visualisation, the inside of a reference architecture model becomes a good choice for CPS interoperability [17,18]. A new proposal to add a semantic layer inside the IEEE 1451 permits it to achieve the framework layer and ensure the semantic level of interoperability required for DT development. 

The present paper highlights the discussion about interoperability in the DT communication layer, and develops an interoperable DT between two different standards, the IEEE 1451 and the IEC 61499. At this moment, the syntactical level of interoperability during the communication was achieved between both standards. However, to reach the semantic level, the IEC 61499 needs to employ a framework, such as OPC UA. The main contribution is a proposed improvement of the IEEE 1451 standard with a semantic layer based on ontology and vocabulary that allows for removing the framework layer from IEC 61499. It enables the IEEE 1451 standard to fulfil the requirement for developing an interoperable DT. The experimental setup acquires data from the physical sensor, sending it in real-time to its digital representation, using the two-way communication required for a DT for monitoring and data visualisation.

The remainder of this paper is structured as follows. Section 2 shows the shop floor digitalisation concept, following the concepts, standards, and protocols used to build a Digital Twin. An interoperable Digital Twin is presented in Section 3, based on the IEEE 1451 family of standards and the IEC 61499 standard. Section 4 describes the implementation of a digital twin based on the semantic layer proposed for the IEEE 1451 standards to visualise and monitor a prototype system. Finally, the discussion and conclusion are in Section 5.

## 2. Digital Twin Background

A Digital Twin enables the representation of an asset from the physical world into the digital world. Grieves expressed the generic definition of a Digital Twin in 2003 [8]. However, the concept of having a representation of an asset was developed by NASA in the early 1960s for the Apollo mission [6]. In 2012, NASA presented a detailed definition of a DT [19]. Since the initial report was delivered, many other authors provided alternative definitions, such as “the cyber layer of CPS, which evolves independently and keeps close integration with the physical layer” [20]. Negri, Fumagalli, and Macchi [21] defined the DT as “a virtual and computerised counterpart of a physical system that can exploit a real-time synchronisation of the sensed data coming from the field and is deeply linked with Industry 4.0”. To many others summarised in [6,11], the DT definition is related to CPS. However, it is difficult to classify what is or is not a Digital Twin because the definitions are ambiguous [8]. 

Three conceptual representations for communication in a DT are very similar: Digital Model, Digital Shadow, and Digital Twin. The Digital Model does not have real-time communication between the physical and digital parts. A Digital Shadow has unidirectional communication between the physical and real-time digital representation. The DT has bi-directional communication from the physical to digital and from digital to real-time physical [5,22,23].

There is no consensus on DT development, and each author introduces a different concept. However, the stages of designing and developing a DT are as follows: mirror the physical into the digital world; monitor and control the DT monitoring; model and simulate the DT from the simulation result of data; federate the DT to optimise the complex objects and interoperated Federated DTs; and, finally, act autonomously, recognising and solving problems in the federated DTs [24].

Author Fuller et al. [9] define the following domains for developing a DT: application, middleware, networking, and object. The application domain consists of the model architecture and visualisation, software and APIs, data collection and pre-processing. The middleware domain comprises stage technology and data processing. Network domains comprise communication technology and wireless communication. Object domains include the hardware platform and the sensor technology.

In [6], the authors Liu et al. conclude that a DT needs to be individualised, being a closer part of its physical representation. With high fidelity, a DT can simulate the behaviour of its physical counterpart as perfectly as possible. Updates at the DT must occur as soon as the physical part is updated, and the communication should be in real-time, with low latency. Finally, controllable changes in one part must be reflected in the other as quickly as possible. For Tao et al. [7], there are five layers of DT modelling: physical and digital parts, data, connection, and service modelling. 

In the industrial sector, predictive maintenance is a possible application. Fault detection, state monitoring, performance prediction, and virtual testing of a new prototype are other potential applications [6]. DT development costs continue to be high, and therefore the technology is employed mainly by large enterprises, such as GE, Siemens, Microsoft, IBM, Bosch, and Tesla. They simulated and predicted the process before the object’s development was able to increase effectiveness inside the large industrial companies by ten per cent [22]. 

The reference architecture models were developed as a general guideline to manage and standardise components inside the industrial environment. The Industrial Internet Reference Architecture (IIRA) for IIoT and the Reference Architecture Model Industry 4.0 (RAMI 4.0) for the I4.0 have received attention as reference architecture models. IIRA was developed by the Industrial Internet Consortium (IIC) and focuses on conceptualising, designing, and implementing architecture in the industrial sector (manufacturing, transportation, energy, and healthcare), from the sensors on the shop floor to the business decisions based on viewpoints and the ISO/IEC/IEEE 42010 standards [25]. RAMI 4.0 was developed by the German Electrical and Electronic Manufacturers Association (ZVEI), and is focused on holistic and adaptive industrial automation and production system architecture focused on manufacturing, as well as defining a structure from sensors to the business decision based on the IEC 62890 standard [26]. Both standards share the same goal of converging the physical and digital worlds, and can interoperate between them at the communication level [27].

The development of a DT can integrate many standards established by different organisations. The ISO 23247-1 ISO/TC 184/SC 4 Industrial data were generated by provision of a framework for DT development. Additionally, associations were formalised for DT development, such as the Industrial Digital Twin Association (IDTA), the Digital Twin Consortium (DTC), the IEC and ISO Joint Technical Committee (JTC 1), the Emerging Technology and Innovation (JETI), and the Change2Twin Consortium in Europe [28]. However, it is challenging to build a DT because many different assets, systems, and processes need to be virtualised, sometimes in a very complex manner, to fulfil the DT requirement, which is composed of three layers: physical, digital, and cyber [22,29]. Standardisation is essential to provide guidelines to interconnect all the physical assets, with its DT representation, crossing the different domain areas [8]. The standards for DT development are [28,30]:

- Reference architecture models: RAMI 4.0 and IIRA.
- Frameworks: IEEE P2413, ISO 23247-1 ISO/TC 184/SC 4, oneM2M, Fiware, OPC UA, MTConnect standard (ANSI/MTC1.4-2018), AutomationML, ISO 15745, ISO 23247, IEC/TC 65, and IEC 61499 tools (NxtStudio and 4DIAC).
- Visualisation and Simulation: Computer-aided design (CAD), ISO/PAS 17506:2012 (COLLADA) modelling tools (Plant Simulation, Demo 3D, nxtHMI, Modelica, three.js, Babylon.js, iModel.js, and Visual Components) [31]. Artificial intelligence (AI) and machine learning (ML) (supervised, unsupervised, and deep).
- Cloud Computing: ISO/IEC TR 22678:2019, ISO/IEC TR 23186:2018, ISO/IEC 19086-2:2018, ISO/IEC 19941:2017, ISO/IEC 19944:2017, ISO/IEC 19086-3:2017.
- Security: IEC 62443, ISO/IEC JTC 1/SC 27, ISO 13849, ISO/IEC TS 33052:2016, ISO/IEC 27017, ISO/IEC 27002, NIST SP 800-82, CSF ISO/IEC TS 33052:2016, blockchain (IEEE 2144, IEEE 2418.2, IEEE 2418.10, and IEEE 3801-2022), and RSA (RFC 3447).
- Communication: Transmission Control Protocol (TCP), User Datagram Protocol (UDP), Hypertext Transfer Protocol (HTTP), Message Queuing Telemetry Transport (MQTT), Extensible Messaging and Presence Protocol (XMPP), Constrained Application Protocol (CoAP), Application Programming Interface (API), Extensible Markup Language (XML), Turtle, Resource Description Format (RDF), JavaScript Object Notation-Linked Data (JSON-LD), Web Ontology Language (OWL).
- Physical sensors and actuators: wireless communication (Wi-Fi, Bluetooth, ZigBee, LoRA, and 5G) wired (POWERLINK, UART, SPIO, I2C, Modbus, and CAN), IEEE 1451, and IEEE 2888.

**Figure 1:**
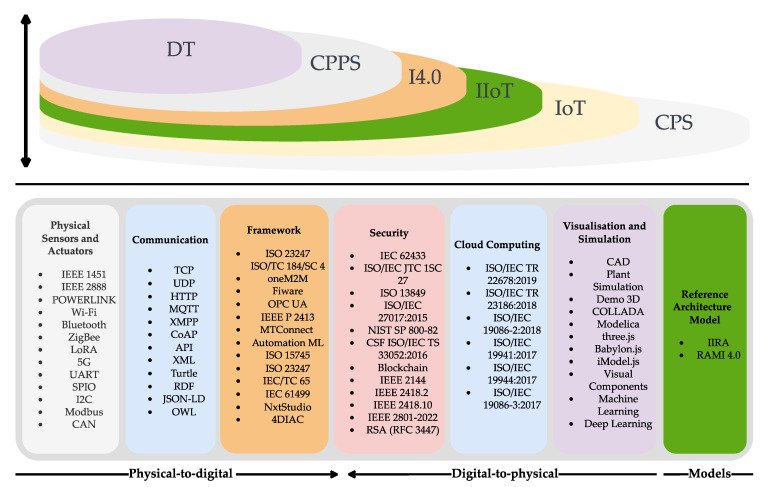
Concepts and standards from physical-to-digital, digital-to-physical, and reference architecture models.

The representation of how the concepts and standards are organised is presented in Figure 1.

Interoperability is one of the main challenges in developing a new DT to interconnect different standards and devices [32,33]. Inside an industrial network communication, many standards and protocols can be employed to promote interoperability. The employment of those standards depends on the application, being a real industrial scenario characterised by different requirements. Solutions for interoperability in the industrial communication layer have been proposed. Scanzio, Wisniewski, and Gaj [34] developed a survey providing solutions for industrial communication based on requirements for industrial applications employing wireless and wired communication technology. In the paper [35], Wollschlaeger, Sauter, and Jasperneite developed a survey about the advances in the technology for industrial communication, employing Ethernet time-sensitive networking and 5G in industrial automation. The authors also present a timeline of the technologies utilised in industrial communication since it started in the 1970s, and discuss the future of industrial communication, such as the industrial communication levels, from the physical sensor to the business functions. Gonzalez et al. [36] wrote a survey about the OPC UA communication acting as middleware inside the industrial environment as a possible solution for the industrial interoperability problem based on its standardisation.

**Figure 2:**
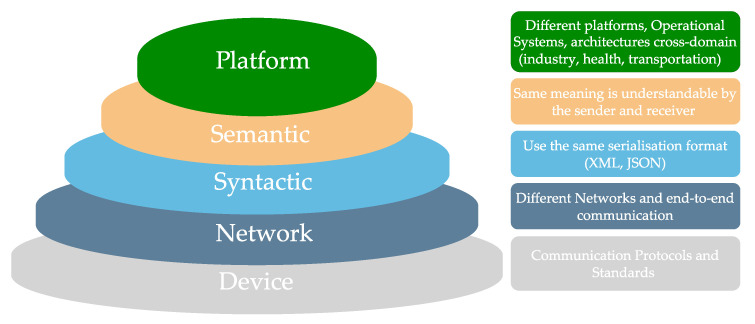
I4.0 Levels of interoperability.

A DT needs to achieve different levels of interoperability, including, at the syntactical level, data from the same serialisation format. On top, there is the semantic level for transferring data ambiguously, being the goal for a DT achieved by employing a framework or employing an ontology, vocabulary, or linked data [14,15]. Figure 2 shows the interoperability levels for the industry. 

There are works employing DT concepts inside the industry. Authors Ding et al. [37] developed a framework for the DT in CPPS, the DT-CPPS, composed of the Physical Shop Floor (PSF) and the Cyber Shop Floor (CSF) and configurable with the bi-directional data flow. It allows synchronisation, interoperability, control, and simulation in a smart factory environment. Appling the RAMI 4.0 Asset Administration Shell (ASS) concept, the author in [32] developed a DT focused on interoperability for the information exchange based on an HTTP RESTful API and files to achieve the syntactical and semantical levels of interoperability. For mass personalisation in the I4.0, the authors Aheleroff, Zhong, and Xu [38] designed a DT reference model as an abstract framework for physical communication, digital, cyber, and application. Sjarov et al. [39] developed the Design for Interoperability (DfIOp) framework for DT and I4.0 considering the interoperability levels and implementation methods, but with further analyses needed for future works.

The IEEE 1451 family of standards is elaborate, focusing on the transducers (sensor and actuator) management. It is composed of IEEE 1451.0 as its main standard, describing the Transducer Interface Module (TIM) and the Transducer Electronic Data Sheet (TEDS). The transducers are connected to the TIM and described by the TEDS, which stores all the necessary information about the transducer (e.g., manufacturer, type, geolocation, calibration, and so forth). The communication from the transducer to the external network is responsible for the Network Capable Application Processor (NCAP) described in the IEEE 1451.1, which is connected wired or wirelessly to the TIM. The NCAP receives the messages from a user’s application, converts them into a command sends the command to the TIM. The TIM answers the NCAP, which converts the response and sends for the user’s application [40].

The IEEE 1451 was developed to manage transducer operation and communication and find applications in DT development. Song et al. [35] developed the universal CPS environment for federation (UCEF) by IEEE 1516 to model and simulate distributed processes. The IEEE 1451 developed a real smart sensor in the field. Three new federates were introduced: IEEE 1451 smart sensor DT Federate (DTF), IEEE 1451 DT Tester Federate (DTTF), and the federation experimenter manager. Mitterer and Zangl [41] designed a DT controlled by the NCAP from the IEEE 1451.1 standard for communication through an API, and the virtual TEDS from the IEEE 1451.4 standard to design, prototype, simulate, and test a robot arm in a real environment. A DT approach based on the IEEE 1451 introducing the Health Electronic Data Sheet (HEDS) is presented by [42]. Shakil et al. The authors of [17] interoperate the IEEE 21451 with OPC UA to develop an interoperable DT creating the representation of IEEE 1451.0 devices in the OPC UA framework. 

The IEC 61499 was developed for industrial automation and management. The communication occurs by client-server or publish/subscribe communication, employing the framework OPC UA, which is also a standard used for DT development. In [43], experiments were developed in the laboratory for a production system with DT built with the IEC 61499, NxtControl software from NxTStudio, communicated by UPC UA and visual component to virtualise the DT [44]. Jhunjhunwala, Atmojo, and Vyatkin [45] built a DT using the IEC 61499 and 4DIAC software for cloud computing, utilised the cloud VM from the node, and built a web server and a web page for visualisation. The test developed was the control of an LED placed on a Raspberry remotely through the IEC 61499 virtual controller. 

This paper aims to reach the syntactic level of interoperability between the IEEE 1451 and IEC 61499 and provide a semantic solution for the IEEE 1451 to achieve the semantic level, turning it into a complete framework for DT communication.

## 3. Development of a Digital Twin Based on the IEEE 1451 Standards

The IEEE 1451 family of standards was developed as a smart transducer interface, defining a set of functionalities independent from the physical transducer communication with its management by common functions, communication protocols, and descriptions. The family of standards is composed of the IEEE 1451.0 as the core standard. This standard defines the Transducer Interface Module (TIM) connected to the transducer (sensor, event sensors, and actuators). It describes the interface to the transducer, the signal condition, and conversion (analogue-to-digital and/or digital-to-analogue). Each transducer has its Transducer Electronic Data Sheet (TEDS) to store the transducer’s information, operation, and data conversion. It communicates by its TransducerChannel (TC) to receive commands. An API controls and manages the transducer through the internet by writing or reading commands from a TIM [40]. The API is defined and utilised by another member of the standard, the IEEE 1451.1, the Network Capable Application Processor (NCAP).

An NCAP defines and implements the services to connect to the TIM and manage the transducers. The Discovery Service is utilised to discover a new TIM and its transducers. The Transducer Access Service enables access to the TC inside the TIM to read and write commands. The TC information is stored in the TEDS and is read and written by Transducer Access TEDS. The Event Notification Service allows the setting and monitoring of events triggered by the sensors or event sensors inside a TIM.

**Figure 3:**
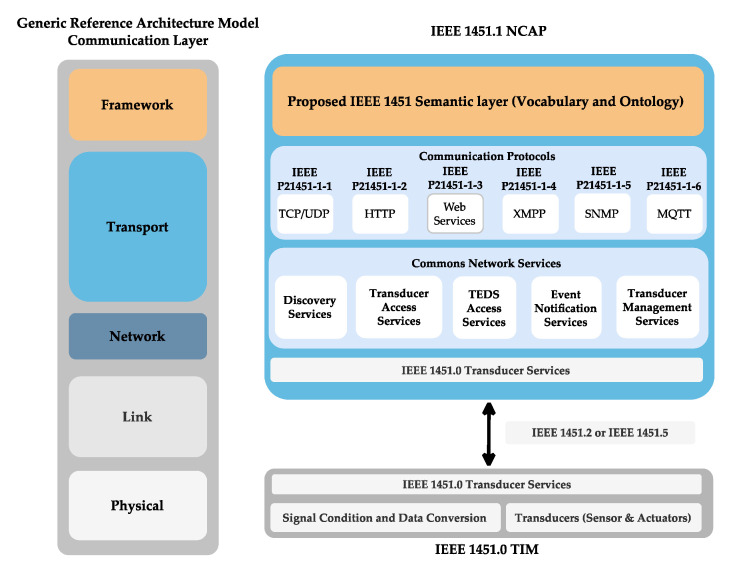
IEEE 1451 family of standards and the proposed semantic layer.

Moreover, the Transducer Management Service enables the sending of commands to the transducers. Internally, the NCAP is composed of classes in a hierarchical order. The NCAP is connected to the TIM by the IEEE 1451.2 if wired, or by the IEEE 1451.5 if wireless, using commands written and read by a set of octets. To allow the transducer’s management through the internet, one of the protocols (TCP, UDP, web services, XMPP, SNMP, or MQTT) is supported by [46]. The NCAP can be employed inside a reference architecture model to achieve a syntactic level of interoperability [18]. To accomplish a semantic level, it needs to use a framework (OPC UA, oneM2M, and others) [15]. To allow the IEEE 1451 to interconnect semantically, the IEEE 1451 semantic layer was proposed based on a vocabulary and ontology providing semantic communication and allowing the IEEE 1451 to fulfil the communication layer of a reference architecture model, as shown in Figure 3. 

**Figure 4:**
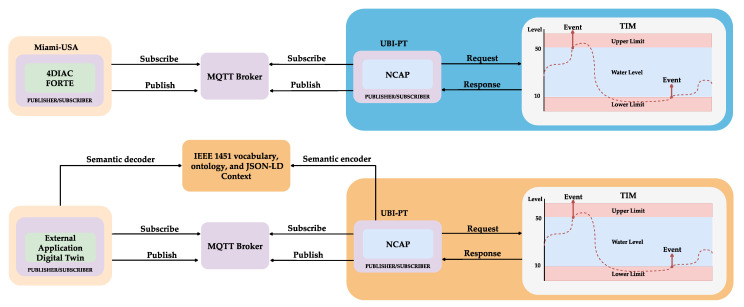
Conceptual scenarios.

A DT environment for water monitoring and status visualisation uses two configuration modes. First, the IEEE 1451 standards interoperate with the IEC 61499 standard, which promotes a semantic level of interoperability by employing the OPC UA framework. The second configuration eliminates the framework layer and promotes semantic interoperability by a proposed semantic layer inside the NCAP from the IEEE 1451 standards, as presented in Figure 4. 

### 3.1. TIM Development

Developing a sensor network that complies with the IEEE1451.0 standard requires knowledge of the entire standard. Creating it by hand demands a high volume of work, and any change in it requires revising of all the development work. A suite of tools is accessible online at http://iml.ubi.pt/ieee1451, designed to minimise new TIM development, test, and validation work. Those tools were developed to rapidly increase the adoption of the IEEE 1451 standards. The TIM and TEDS editors allow the development and configuration of a TIM with its corresponding TEDS. After the setup, the system lets the user download the code using XML or TXT formats. After successful compilation and building, the executable code is transferred to the MCU onboard the boards supported. Validating the code is possible using the TIM Validator [16].

**Figure 5:**
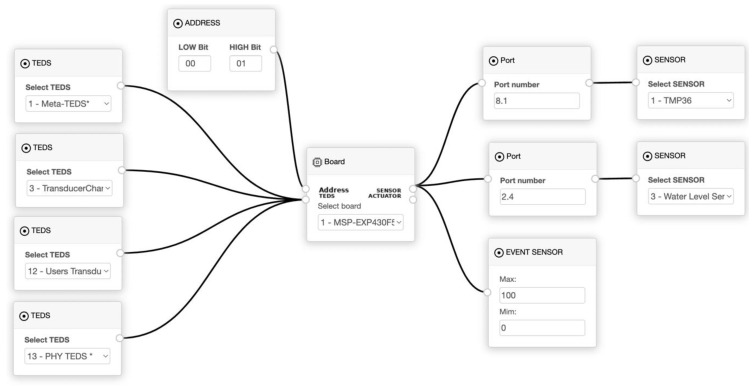
TIM was developed using the TIM Editor developed at the UBI laboratory.

A TIM contains the four mandatory TEDS, specified by the IEEE 1451.0 standard. Meta TEDS stores information about the TIM, such as localisation, manufacture identification, year of manufacturing, and number of TCs. The second mandatory TEDS is the TransducerChannel TEDS that stores information for each TC defined inside the Meta TEDS, such as the calibration key, type (sensor, event sensor, and actuator), the physical units for conversion, the range of transducer operation, the sampling modes, and the buffer attributes. A name can be defined for the TC by UsersTransducerName TEDS, the fourth one being the Physical TEDS. The Physical TEDS stores information about wireless communication, such as radio type and version, throughput, latency, encryption, and authentication. The TIM Project Editor allows visual management of the TIM project, as presented in Figure 5.

For this setup, an MSP430F5529 board from Texas Instruments implements a TIM using the Code composer Studio and C language, with two sensors, the temperature and capacitive water level sensors. An event sensor detects when the water reaches the limit level. The order of the TCs is:

- TC 3: temperature rate sensor.
- TC 5: water level sensor.
- TC 6: event sensor water level upper limit.
- TC 7: event sensor water level lower limit.

**Figure 6:**
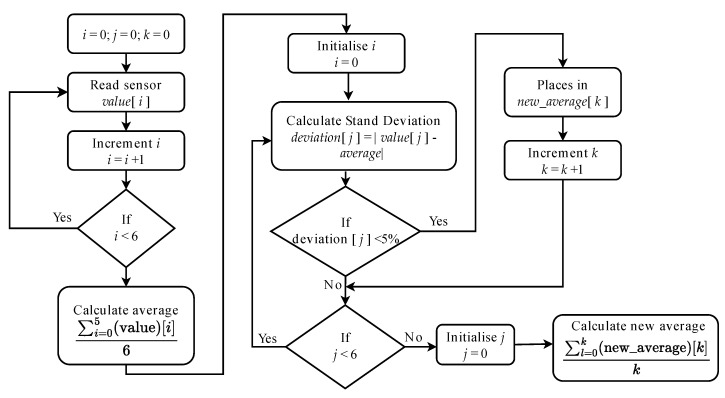
Water levels acquisition algorithm.

When the water reaches an upper or lower limit, it generates an event that outputs the value one from the TC. The water levels algorithm is shown in Figure 6. It reads the water level sensor and computes its standard deviation to calibrate and provide a more accurate sensor read. The values are recorded in the TC’s buffer and read by a command from the NCAP. A sensor TMP 36 from Analog Devices, connected to the MSP430F5529, measures the temperature. 

The level of water detection uses a timer to measure, in microseconds, the charging time of a capacitive sensor through a resistance. The timer starts counting at the charging period. When the voltage of the capacitive sensor reaches 2.44 V, an interruption occurs, and the timer stops counting. The time value changes with the water levels because the capacitance changes with the sensor’s level of immersion in the water, following Equation (1) and shown in Table 1.

*WaterLevels* (%) = 0.717 × *time* − 70.26 (1)

Data obtained with the sensors is stored inside each TC, waiting for the NCAP’s read transducer data segment command. Commands are composed of hexadecimal octets with the TIM ID, transducer channel ID, command class, command function, length, and command dependent, as shown in Table 2.

### 3.2. NCAP Development

The NCAP was implemented by classes using the python language and the Raspberry Pi 3B+. The NCAPBlock, the main class, contains information about the configuration, such as manufacture identification, model number, software version, and the operating system for the NCAP and Block. The block can be utilised and interoperated with other NCAP and NCAPBlocks. The NCAP in network services employs the MQTT protocol to communicate, and there are services to send commands between the NCAP and the TIM.

In the first step, the NCAP sends a command to read the Meta TEDS, TransducerChannel TEDS, UserTransducerName TEDS, and Physical TEDS. The answer is decoded utilising the TLV (Type/Length/Value) format. Based on the information acquired from the Meta TEDS, the NCAP extracts the TC information and its address used to send the commands to the TransducerChannel. The TransducerChannel TEDS contains the information about each TransducerChannel; that is, information on whether it is a sensor or actuator, on the calibration, the units used for data conversion, the value from the TransducerChannel, the operating state, the sampling mode, and on the time sampling. The transducer’s name is retrieved from the UserTransducerName TEDS. The Physical TEDS provides information about wireless communication.

**Figure 7:**
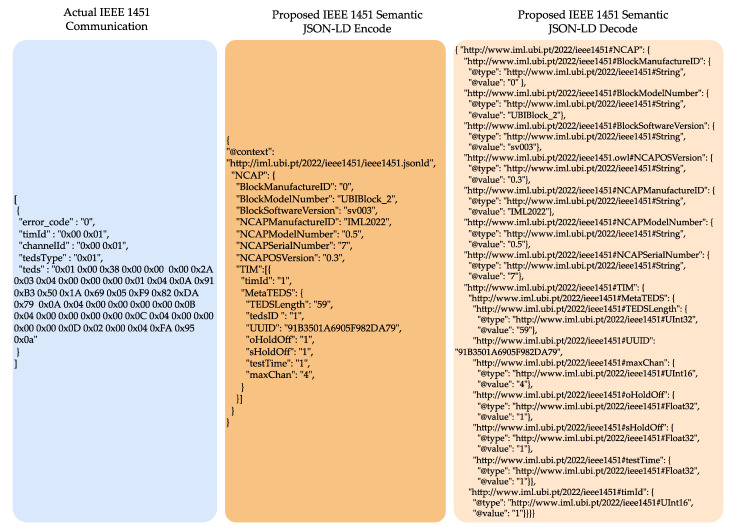
Actual and proposed NCAP semantic communication.

The information can be requested as raw information in the octet format or decoded by the end user. The communication occurs using the publish/subscribe mechanism from the MQTT protocol [47]. It allows the NCAP to achieve the syntactical level of interoperability [48]. The IEEE 1451 requires a semantic service that converts the data semantically into a vocabulary and ontology based on the IEEE 1451 family of standards to reach the semantic level needed for a DT. The development uses the OWL language and the JSON-LD [49] to link the data information from inside the NCAP and decode the information with the receiver (human or machine). With this proposed implementation, the developer does not need deep knowledge of the standard. Before message exchange, the linked data [50] is used to obtain the mandatory information at the requested time, as presented in the Meta TEDS example in Figure 7. The first box (Actual IEEE 1451 Communication) presents the actual implementation of IEEE 1451. The commands are sent and received in octet format. The user must study the standard and TLV (Time/Length/Value) format to know what each octet or octet array represents. For example, the “timId” field. The user must access the IEEE 1451.0 standard documentation and search for what “timId” means in this case: “The number composed of two octets that need to be converted in Uint16 corresponding to TIM’s identification number with value 1”. The proposed semantic layer second box (“Proposed IEEE 1451 Semantic JSON-LD Encode”) is placed inside the NCAP to convert the octet to the format defined by the standard in the “timId” example in Uint16 automatically. It is encoded using the JSON-LD format that uses JSON format, which is widely used in internet communication with Linked Data. This allows for the creation of a context for the communication. The receiver of this communication, the third box, “Proposed IEEE 1451 Semantic JSON-LD Decode”, receives the data in JSON format and decodes the data using the JSON-LD. Based on the linked data, the receivers know that the “timId” is a Uint16 with the value “1” without the need to read the IEEE 1451 standard documentation; additionally, the vocabulary is available to consult the terms used during the communication. The files and documentation are available at http://iml.ubi.pt/2022/ieee1451.

## 4. Testing the Interoperable Digital Twin

Interoperability is essential in shop floor communication. A water monitoring scenario was used to test the interoperability in a DT. This scenario allows real-time monitoring of the water level and temperature to establish a DT for visualisation and monitoring. Two tests were created: the first provides interoperability between the IEEE 1451 and the IEC 61499 standards. The IEEE 1451 was used to acquire data, and the IEC 61499 was used to visualise the data and control, and provide the syntactic by the MQTT protocol supported by this 4DIAC tool. The second test was developed using the new proposed semantic layer from the IEEE 1451 for the visualisation and control of the DT.

The IEC 61499 standard was developed for industrial distributed automation systems focusing on portability, reusability, reconfigurability, and interoperability. It provides a generic model composed of processes and communication networks based on the functional blocks (FBs) encapsulating its functionalities. An FB has the event entry on its head to control its execution, which is processed on its body part, generating an output event [51]. The Eclipse 4DIAC tool was developed to be a cross-domain and development environment based on the IEC 61499. The 4DIAC FORTE is a portable runtime implementation of this standard designed to run in small devices (16/32 Bit) that allow execution of all the FBs defined in the standard. It supports different communication protocols (e.g., MQTT, HTTP, OPC UA), boards (e.g., Raspberry Pi (Raspberry Pi Trading Ltd., Cambridge, UK), BeagleBone (Texas Instruments, Dallas, TX, USA), and so on), operational systems (e.g., Windows (Microsoft, Redmond, WA, USA), Linux based), and Programmable Logic Controllers (PLCs) (e.g., Bachmann (Hamilton, Bermuda), MicroControl (Troisdorf, Germany), Wago (Minden, Germany), Siemens (München, Germany)) [52]. 

PLC supports different communication protocols. In this paper, MQTT was used, since it is supported by both IEEE 1451 and IEC 61499 standards. It should also be mentioned that another alternative for publish/subscribe is the usage of client-server, which on the one hand, is supported by two standards, and on the other hand, requires more computational complexity.

It should be noted that, although MQTT is the fastest and cheapest messaging protocol for IIoT applications, the broker can be a single point of failure, which could potentially shut down the whole network communication. However, to improve reliability, MQTT supports a bridging mechanism by adding redundancy to the broker at the quality of service (QoS) level. 

**Figure 8:**
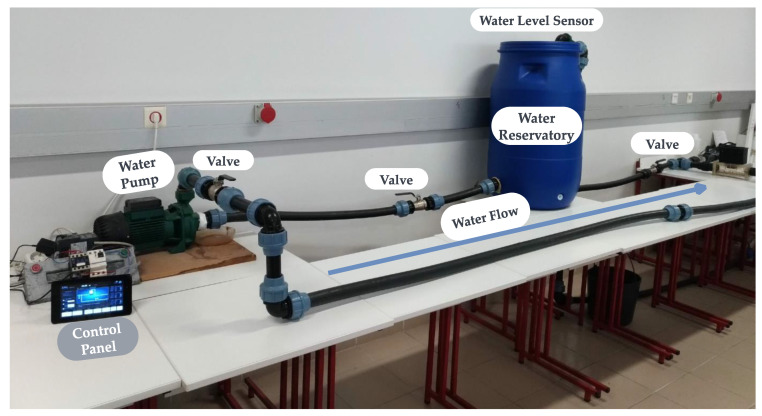
Setup in the UBI laboratory.

The first DT interoperable scenario was established between the Miami University-Ohio (United States), and the University of Beira Interior (UBI) (Portugal). The IEEE 1451 was implemented inside the IML-UBI laboratory, and the 4DIAC application was developed at Miami University. The water monitoring prototype setup is presented in Figure 8.

### 4.1. Interoperable IEEE 1451 and IEC 61499 Digital Twin 

**Figure 9:**
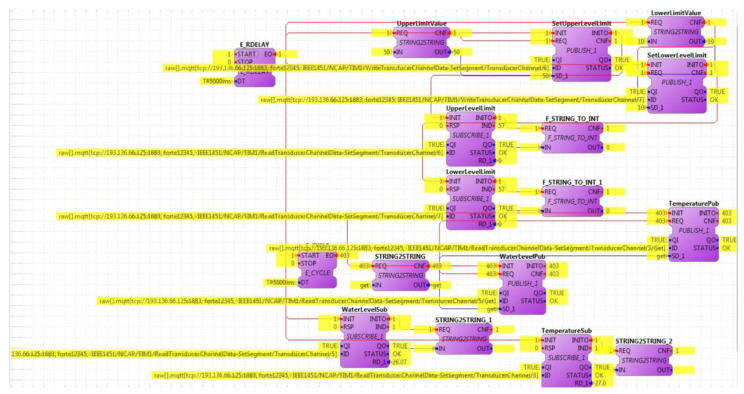
IEC 61499 Function Blocks receiving and visualising data inside the Eclipse 4DIAC from the IEEE 1451 standards acting as an interoperable Digital Twin.

This setup test used the actual communication defined at the IEEE 1451 standards. The 4DIAC software was used to implement the IEC 61499 and send and receive message requests from the IEEE 1451 implemented at the UBI. The 4DIAC and the NCAP are subscripts to the topics in the MQTT broker. The NCAP sends the command to request the TEDS to the TIM using octet hexadecimal format. The TIM answers the NCAP with the TEDS in the same format. The TEDS are stored inside the NCAP. The TEDS are decoded and sent when it is requested. Based on the information stored inside the Meta TEDS, the TransducerChannels address was read. The address enabled the reading and writing of data in a TransducerChannel. There were two commands from the 4DIAC to the NCAP to set the upper and lower limits for the water monitoring alarms. There were two commands for data acquisition (water level and temperature). The 4DIAC subscripts to the related topics and receives the data acquisition. Figure 9 presents the IEC 61499 function blocks inside the 4DIAC software during real-time communication. The function blocks implementing the MQTT publisher were utilised to set the limits for the Digital Twin, with a lower-level limit of 10% and an upper-level limit of 50%. The lower and upper alarms were subscripted to the MQTT topics, followed by the water level and temperature to visualise and monitor the system. The yellow text highlighted in the figure means that all the connections were properly configured and working normally. This text overleaps the initial configuration inside the 4DIAC, making the data visualisation difficult.

The limitation of this implementation is that the user needs to know about the IEEE 1451 family of standards and the IEC 61499 standard. The previous implementation achieves a syntactic level of interoperability. The IEEE 1451 standard does not support a framework for communication in the standard specification. However, it supports communication protocols, such as MQTT and HTTP [46].

In a preview work [18], the author studied network communication for a similar setup. It compared the MQTT and HTTP protocols for industrial communication. The preview paper concludes that the MQTT was faster, even for cross-continental communication. The internal network latency was 43 milliseconds using MQTT and 67 milliseconds for HTTP. External communication from UBI-Portugal to Miami University USA occurred in 41 milliseconds using MQTT, and in 265 milliseconds using HTTP. Being the MQTT acceptable for scale reading according to [53] determines the latency of 100 milliseconds for this application. 

It is necessary to implement a framework outside of IEEE 1451 scope to achieve the semantic level of interoperability. The 4DIAC tools support the communication protocols and the framework, which can receive syntactic messages from IEEE 1451. After the procedure, the composition of the message using a framework supported by the 4DIAC is the OPC UA [52]. This interoperation mechanism has a complex and slow development, making it difficult to communicate and collaborate with different standards. It is already complex to implement a standard and to integrate it into a framework that was not originally developed to work with it becomes a difficult task. A new semantic layer was proposed based on the IEEE 1451 to remove the complexity of implementation and speed up the DT development, allowing this family of standards to support semantic communication, supplying the framework layer and achieving all the requirements for DT communication. 

### 4.2. Interoperable IEEE 1451 Semantic Digital Twin

The second scenario proposed including a semantic layer in the IEEE 1451 standards. The NCAP requested information from the TIM and stored it inside the NCAP. Information was serialised using the JSON-LD format. A vocabulary and ontology were developed using the OWL language based on the IEEE 1451 standards. This approach created a JSON-LD context inside the NCAP to encode the data semantically using the linked data by Uniform Resource Identifier (URI) to the external file containing the information. When information about the system was required, a new JSON-LD file was encoded and sent over the MQTT protocol. The linked data let the receiver decode the message using the JSON-LD context. In this way, the application (user/machine) did need to know the IEEE 1451 standard before it could retrieve the information about the NCAP, TIM, Meta TEDS, and TransducerChannel when it was required.

**Figure 10:**
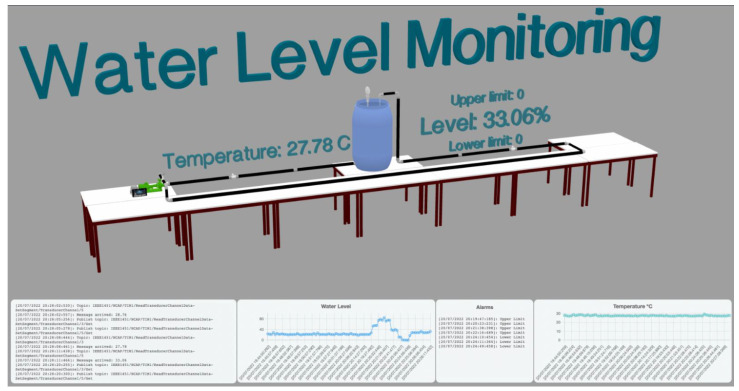
Interoperable Digital Twin based on the IEEE 1451 semantic communication.

The JSON-LD allows the encoding of data with RSA encryption or the signing of data using blockchain. The message received has MQTT topics that users or machines can subscribe to read or publish to retrieve data from the TransducerChannels. The graphical model allows the simulating and testing of the data without a real scenario, which improves the development and testing. This DT was utilised to create the 3D model of the three.js tools, a JavaScript framework presented in Figure 10.

In this DT implementation, the application requests information from the NCAP. Using the IEEE 1451 JSON-LD context, the NCAP serialises the system information and returns it to the application. The application decodes the JSON-LD, obtaining the data from the file that contains the IEEE 1451 ontology information. The application subscribes to the MQTT topics provided in the JSON file, and starts receiving the system’s data. The data are the temperature, water levels, and the upper and lower alarms. 

The benefit of this implementation is that the information about the IEEE 1451 is stored and requested when needed, reducing the complexity of the DT development and serving a semantic communication requirement for DT communication using linked data. The vocabulary and ontology developed based on the IEEE 1451 enable the existence of a centralised database with the information on IEEE 1451 standards. Developers and machines can use this database to consult the information about the standard, such as the description of components and datatypes defined in the standard, avoiding any misunderstanding of concepts during the implementation. Moreover, it allows other developers to use the information to build their ontologies using the data stored inside the file at the UBI laboratory. 

The proposed semantic layer turns the IEEE 1451 into a complete framework for transducer management, from the physical sensor to the semantic level of communication. This supplies the need for semantics for the TD communication [11,39,54]. 

The disadvantage is that the industry’s IEEE 1451 is not yet widely employed, and is under review as the IEEEP 1451.0.

It is important to mention that all the tools utilised are open source and available online or to the community, including the python language, Eclipse 4DIAC, Raspberry Pi, IEEE 1451 ontology and vocabulary, the TIM TEDS manager developed at UBI, Mosquitto MQTT broker, and three.js for the 3D visualisation.

## 5. Discussion and Conclusions

The Digital Twin is a technology employed in many different sectors, from general IoT applications to the complex manufacturing process inside Industry 4.0. There are no default frameworks, tools, or methods for developing a DT. The development must meet the system requirements. This paper presents the standards, frameworks, tools, and reference architecture models for DT development, focusing on interoperability knowledge. However, communicating and coordinating different protocols, tools, and standards remain complex tasks in DT development. Standardisation can help ensure DT development can be carried out without erroneously defined concepts [9].

The IEEE 1451 family of standards was developed in the early 2000s to manage transducers, incorporating the TIM and TEDS with transducer information; in the active version of this standard, to interoperate with other systems only was possible at a syntactic level, but a framework was required for the semantic. This was shown in the first interoperable scenario. Semantic allows the DT to communicate ambiguously, which is a requirement for the DT development [6]. Additionally, it may help adapt the IEEE 1451 to more applications in different areas. 

This paper aims to develop an overview of the technologies used for communication during the DT development in the academic and industrial areas, from sensor data acquisition to visualisation with real-time communication. The work discusses interconnecting protocols and standards for the development of an interoperable system, digitalising the shop floor using the IEEE 1451 standards, with other standards on syntactic and semantic levels. 

Based on the new proposed ontology and vocabulary using the link data, the semantic level was reached inside the IEEE 1451 family of standards using the IEEE 1451 context developed by the JSON-LD. The security was placed inside the MQTT protocols, and the JSON-LD implementation allowed the use of RSA cryptography and signed the message with blockchain. It allowed the addition of a security layer inside the IEEE 1451. This communication layer can be utilised inside a reference architecture model. Tools used in TD development are open source and open to the community. Using the information inside the TEDS can reproduce the physical sensor in the digital world. 

This work has some limitations. The communication protocol MQTT is faster than the HTTP, but not reliable using the QoS 0. The 4DIAC tool only accepts the MQTT QoS 0 during the communication. The HTTP can be employed for reliability. The IEEE 1451 does not support all the communication protocols utilised on the older PLCs, such as Modbus, Optomux, CAN, and others. Another limitation is that the work is focused on the industrial environment’s level 0 (sensor and actuator layer). It was not tested with SCADA or other control systems, such as Nxt Studio, which leaves new interoperable scenarios for future work. Another limitation is security. The IEEE 1451 does not provide a security layer. Security is handled by communication protocol or semantic communication solution. Regarding the linked data limitation, as the communication point for the same vocabulary, this vocabulary needs always to be available. Serving other ontologies and applications creates a weak point in case of internet connection failure.

Future research should employ machine learning and artificial intelligence for simulation and prediction. The implementation in a real industrial car manufacturing environment is part of a project that is being developed. 

## Figures and Tables

**Table 1:** Time to load the water level sensor.

Water Level (%)	Time (ms)	Average (ms)	Water Level (%)	Time (ms)	Average (ms)
0	98	97	50	169	168
	97			168	
	95			167	
10	112	114	60	182	181
	114			181	
	116			180	
20	127	127	70	196	196
	128			198	
	126			193	
30	139	138	80	210	210
	138			208	
	137			211	
40	153	153			

**Table 2:** Commands from the NCAP to the TIM.

TIM ID		Trans. Channel ID		Class Cmd	Function Cmd	Length		Dep. Cmd
**TransducerChannel 3–Temperature sensor TMP 36**								
0x00	0x01	0x00	0x03	0x03	0x01	0x00	0x00	0x00
**TransducerChannel 5–Water level Sensor**								
0x00	0x01	0x00	0x05	0x03	0x01	0x00	0x00	0x00
**TransducerChannel 6-Water Upper-Level Alarm**								
0x00	0x01	0x00	0x06	0x03	0x01	0x00	0x00	0x00
**TransducerChannel 7-Water Lower-Level Alarm**								
0x00	0x01	0x00	0x07	0x03	0x01	0x00	0x00	0x00

The answer from the TIM to the NCAP’s commands is organised in an array of octets as follows: the first octet represents the success/failure, the second is the length, and the third is the value, for example: “0x01 0x00 0x04 0xXX 0xXX 0xXX 0xXX”.

## Abbreviations

The following abbreviations are used in this manuscript:

3D	Three-dimensional
AI	Artificial intelligence
API	Application programming interface
CAN	Controller area network
CC	Cloud computing
CoAP	Constrained application protocol
CIFB	Communication interface function blocks
CPS	Cyber-physical systems
CPPS	Cyber-physical production systems
DT	Digital twin
FB	Function block
HTTP	Hypertext transfer protocol
I2C	Inter-integrated circuit
I4.0	Industry 4.0
IEC	International electrotechnical commission
IEEE	Institute of electrical and electronics engineers
IIoT	Industrial Internet of Things
IIRA	Industrial internet reference architecture
IML	Instrumentation and measurement laboratory
IoT	Internet of things
ISO	International organization for standardization
JSON	JavaScript object notation
JSON-LD	JavaScript object notation linked data
M2M	Machine to machine
ML	Machine learning
MQTT	Message queue telemetry transport
ms	Milliseconds
NCAP	Network capable application processor
OPC UA	Open platform communications unified architecture
OSI	Open systems interconnection
OWL	Web ontology language
PLC	Programmable logic controller
PT	Portugal
QoS	Quality of service
RAMI 4.0	Reference Architecture Model for Industry 4.0
RSA	Rivest–Shamir–Adleman encryption
SIFB	Service interface function Blocks
SMTP	Simple Mail Transfer Protocol
SSN	Semantic Sensor Network
TC	TransducerChannel
TEDS	Transducer electronic data sheet
TIM	Transducer interface module
TVL	Type/length/value
UART	Universal asynchronous receiver–transmitter
UBI	University of Beira Interior
URI	Uniform Resource Identifier
USA	United States of America
XML	Extensible Markup Language
XMPP	Extensible Messaging and Presence Protocol
